# Regulatory framework of human germline and heritable genome editing in China: a comparison with the United States and the United Kingdom

**DOI:** 10.1093/jlb/lsaf007

**Published:** 2025-05-13

**Authors:** Yawen Zou, Yanting Li, Yingshi Tao

**Affiliations:** Institute of Humanities, ShanghaiTech University, No. 393 Huaxia Middle Road, Pudong District, Shanghai 201210, China; Academy for Advanced Interdisciplinary Studies, Peking University, No. 5 Yiheyuan Road, Haidian District, Beijing 100091, China; School of Marxism, Hunan University, No. 2 Lushan South Road, Yuelu District, Changsha 410082, China

**Keywords:** China, CRISPR, human germline genome editing, human heritable genome editing, regulatory framework

## Abstract

Genome editing technology is rapidly advancing and has generated significant controversy, particularly in the field of human heritable genome editing, while also presenting vast potential applications. Following the He Jiankui incident in 2018, there was a global call to reinforce the regulatory frameworks governing human germline and heritable genome editing. China’s existing regulatory framework for human genome editing has improved with several laws enacted and updated, but there are shortcomings. These include overlapping responsibilities of multiple governing agencies and limited involvement of patient groups and the public in the legislative process. By drawing insights from regulatory agencies, legislation, and multigroup participation from abroad, especially in the United Kingdom and the United States, we can compare the differences between China and foreign countries and help China enhance its regulatory framework based on international practices. This article proposes recommendations for enhancing China’s regulatory framework, such as clarifying the responsibilities of agencies, updating policies in a timely manner, strengthening bioethics education and training, and emphasizing the need for a forward-looking, balanced, meticulous, and adaptable regulatory approach.

## I. INTRODUCTION

In the past two decades, genome editing technology and gene therapy have significantly improved efficiency and precision compared with previous methods. In March 2023, the organizing committee of the Third International Summit on Human Genome Editing issued a statement, noting significant progress in somatic genome editing. It highlighted that the current high cost of gene therapies is unsustainable and emphasized the urgent need for a global commitment to making these treatments affordable in an equitable manner.^1^ As of December 8, 2023, just 11 years after the emergence of CRISPR/Cas9 genome editing technology, the Food and Drug Administration (FDA) has approved the first CRISPR/Cas9-based gene therapy, Casgevy, which is used to treat sickle cell disease in patients aged 12 years and older, marking a significant breakthrough in gene therapy.^2^

This article adopts the definitions of heritable genome editing and germline genome editing from Baylis et al. (2020):

Heritable genome editing involves the transfer of genetically modified embryos to a uterus to initiate a pregnancy that would result in the birth of a child with a modified genome. Like heritable genome editing, germline genome editing also involves making genetic modifications to gamete precursor cells, eggs, sperm, or early-stage embryos in the laboratory; unlike heritable human genome editing, any resulting genetically modified embryos are not used for reproduction.^3^

The potential in human heritable genome editing includes the prevention of genetic diseases, addressing infertility, combating infectious and chronic diseases, enhancing preclinical animal models, and facilitating the development of new vaccines and drugs.^4^ Consequently, heritable genome editing has profound implications for various aspects of society, attracting significant attention from both academia and the wider public.

Currently, in many countries, laws explicitly prohibit human heritable genome editing, and no country has officially endorsed this type of clinical trial. However, for couples who suffer from genetic diseases and are very likely to pass them on to their offspring, this technology holds the potential to enable them to conceive healthy, genetically related children. Nonetheless, it requires an effective and adaptable regulatory framework. The absence of such a framework, coupled with the uncertainties surrounding genome editing technology, poses risks and potentially severe consequences. Michael Morrison from the Center for Health, Law, and Emerging Technology at Oxford University noted that achieving consensus in this domain is a challenge, and it is difficult to predict when consensus can be achieved. Morrison also argues that considering genome editing technology within the broader context of the biotechnology industry is crucial. Overly stringent measures may curtail the growth of the biotechnology industry, thereby impeding national competitiveness.^5^ At the international level, there are significant differences in regulatory agencies across different jurisdictions, influenced by their cultures, legal systems, government models, and ethical perspectives. China strengthened its supervision of human germline and heritable genome editing following the He Jiankui incident. This article focuses primarily on China’s regulatory framework while drawing on relevant experiences from other regions, particularly the United States (US) and the United Kingdom (UK).

This article begins by providing a literature review of the history of human genome editing and the ethical controversies surrounding it. Then, it investigates three key components of the regulatory framework: regulatory agencies, policies (including laws, regulations, and guidelines), and the involvement of various social groups. For each component, this article investigates the current state and issues, experiences outside of China, and recommendations. A detailed examination of the current regulatory framework reveals the need to adopt four key regulatory principles: forward-looking, balanced, meticulous, and adaptable approaches.

## II. LITERATURE REVIEW: HISTORY AND ETHICAL DISPUTES OF HUMAN GENOME EDITING FROM A CHINESE PERSPECTIVE

**Figure 1:**
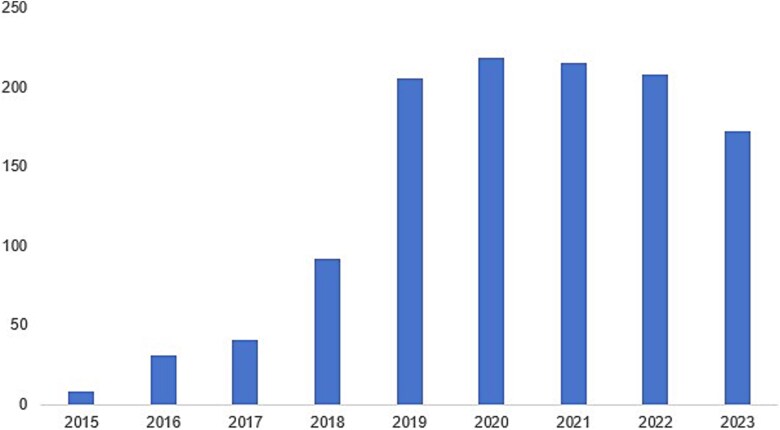
Number of articles on the ethics and regulation of human genome editing in the CNKI database.

The He Jiankui incident in 2018 intensified discussions on the ethics of human genome editing in China. Figure 1 illustrates the number of articles published annually on the topics ‘human + genome editing + regulation’ and ‘human + genome editing + ethics’ in CNKI, the largest database of academic articles in China over the past decade. In 2015, there were fewer than 10 articles on the ethical and regulatory discussions of human genome editing. The number of articles on these topics soared after the He Jiankui incident, increasing to 205 in 2019. Its popularity has slightly decreased since 2021, but still more than 150 articles were published on these topics in 2023.

### II.A. Pre-He Jiankui Era

Before the He Jiankui incident, China’s research on genome editing in humans and nonhuman primates was at the forefront globally, exemplified by several key advancements.^6^ Research on genome editing in human somatic cells or primates is generally considered to be less controversial than research involving human germline and heritable genome editing. In 2014, Nanjing Medical University and Yunnan Primate Biomedical Research Key Laboratory successfully produced the world’s first genome-edited monkey.^7^ In 2016, the journal *Nature* reported the first clinical trial involving genome editing in human somatic cells using CRISPR technology.^8^ Researchers from the West China Hospital of Sichuan University used CRISPR technology to disable the PD-1 gene in T cells as a treatment for patients with metastatic non-small cell lung cancer. By 2023, the clinicaltrials.gov website listed over 80 clinical trials employing CRISPR to address various conditions, including breast cancer, lung cancer, tuberculosis, HIV, β-thalassemia, and sickle cell anemia. More than 20 clinical trials using CRISPR in mainland China have been registered on the website.^9^ For example, Professor Deng Hongkui from Peking University and his colleagues published a study in 2019 on the use of CRISPR to knock out the CCR5 gene in hematopoietic stem cells for the treatment of leukemia, marking the world’s first case of CRISPR-based leukemia treatment. His work earned him recognition as one of *Science* magazine’s top 10 scientific figures of 2019.^10^ Currently, human somatic gene editing is widely applied in basic, preclinical, and clinical studies involving the treatment of cancer and hematological, endocrine, and immune system diseases.^11^

Research involving human germline genome editing had caused significant controversy and ethical debate. In 2015, a team led by Huang Junjiu from Sun Yat-sen University edited human triple pronucleus embryos in the laboratory for the first time. The HBB gene in the genome of these embryos, which were unviable and discarded by hospitals, was edited as basic research for the treatment of β-thalassemia. The publication of this research in the journal *Protein & Cell* attracted significant attention from both the Chinese and international communities.^12^ Although Huang used discarded embryos, Chinese and foreign ethicists had quite different opinions about Huang’s research.

Following Huang’s experiment, a leading Chinese bioethicist, Professor Qiu Renzong expressed his viewpoint that human somatic and germline editing should be permitted while human heritable genome editing and genetic enhancement should be prohibited.^13^ Basic research aims to understand fundamental scientific principles, whereas clinical trials involve human participants to test the safety and efficacy of medical interventions. Therefore, he defended Huang’s experiment on germline genome editing for nonreproductive purposes from an ethical standpoint. Qiu’s view was representative of many ethicists in China.

However, Huang’s experiment split the international scientific community, with most Western scientists holding a negative attitude toward Huang’s research. In 2015, the Science Edition of the *New York Times* published an article titled ‘Scientific Ethical Divide Between China and West’, highlighting the fact that Western scientists had generally abandoned such research at that time. The international community widely believed that China’s regulatory framework for genome editing was too lenient.^14^ The *Nature* and *Science* magazines initially rejected Huang’s submission but later published multiple articles discussing his research. Edward Lanphier, the Chairperson of Sangamo BioSciences, and others, directly condemned Huang’s research in an article titled ‘Don’t edit the human germ line’.^15^ Similarly, Nobel laureate Jennifer Doudna argued that editing the human germline would do more harm than good, stating that there was still much to learn from other biological systems, such as mouse and primate models.^16^ On the other hand, George Daley, a stem cell biologist at Harvard University, noted that Huang’s research adhered to the 14-day norms of embryo research and provided insights into the efficiency of genome editing. Despite this controversy, Huang Junjiu was selected as an important scientific figure in 2015 by *Nature* magazine because he was the first to conduct genome editing in human germline cells.^17^

While the academic community held different attitudes toward human germline genome editing, its members generally condemned human heritable genome editing. In 2015, the First International Summit on Human Genome Editing was held, with the participation of over 3000 scientists from more than 50 countries. Experts from the United States National Academies of Sciences, Engineering, and Medicine (NASEM), the British Royal Society, and the Chinese Academy of Sciences (CAS) served as members of the organizing committee. The Committee, speaking for itself, stated that basic and preclinical research in both human somatic and germline cells was needed and should proceed with appropriate legal and ethical oversight. The clinical applications of genome editing in somatic cells, such as for treating diseases like sickle-cell anemia, were permissible after careful evaluation within existing regulatory frameworks. However, the committee emphasized that human heritable genome editing should be prohibited at present, until there is a consensus in favor of it after resolving the relevant safety and efficacy issues.^18^ In 2017, the NASEM released a report titled *Human Genome Editing: Scientific, Medical and Ethical Considerations*, which suggested that, in the future, human heritable genome editing could be allowed for treating certain serious diseases. However, the NASEM did not advocate for immediate heritable genome editing, nor did it call for a moratorium or prohibition on it. Instead, it emphasized the need for strict oversight and international consensus before any clinical application.^19^

### II.B. Post-He Jiankui Era

However, in 2018, the controversial He Jiankui incident occurred just before the second International Summit on Human Genome Editing held in Hong Kong. He genetically modified the CCR5 gene to prevent the inheritance of HIV from an infected father to his children. The organizing committee of the summit stated that:

the scientific understanding and technical requirements for clinical practice remain too uncertain and the risks too great to permit clinical trials of germline editing at this time. Progress over the last three years and the discussions at the current summit, however, suggest that it is time to define a rigorous, responsible translational pathway toward such trials.^20^

He was widely condemned domestically and globally. In response to the incident, renowned scientists, including Eric Lander, Zhang Feng, and Emmanuelle Charpentier, published an article in *Nature* calling for a moratorium to human heritable genome editing while expressing support for human germline genome editing and clinical applications for somatic genome editing.^21^

Following the He incident, on May 9, 2019, *Nature* published an article titled ‘Reboot ethics governance in China’ written by several leading Chinese ethicists. The article proposed six suggestions for the regulatory framework, emphasizing the need for regulation, registration, monitoring, information dissemination, education, and discrimination prevention.^22^ The authors also discussed 11 essential conditions for human heritable genome editing to enter clinical practice including the need for continuous reassessment of the benefits and risks to health and society. They emphasized that if a society lacks an adequate regulatory framework and that ethical issues have not been addressed, its scientists and doctors are not qualified to engage in human heritable genome editing.^23^ His experiment in China is also considered by some an act of ‘ethical dumping’: those unethical experiments that cannot be carried out in countries with strict ethical scrutiny are transferred to countries with less stringent ethical rules. In December 2019, He was ultimately convicted of illegal medical practices and sentenced to prison for 3 years. He violated *Article 336 of the Criminal Law of the People’s Republic of China*, which prohibits unlicensed medical activities. China has also realized the importance of ethical governance in science and has accelerated legislation in this area. Subsequently, the amendments of the *Civil Code* and the *Criminal Law of the People’s Republic of China* prohibit the implantation of genetically edited human embryos into humans or animals.

In 2020, a study surveyed the policies of 96 countries regarding human germline genome editing. Eleven countries allow such experiments (including China, India, Thailand, the UK, Japan, and the US, which allow experiments with private funds and non-federal public funds such as state or municipal funds in most states). Nineteen countries explicitly prohibit such experiments, and 56 countries have no clear policies. However, no country currently allows human heritable genome editing, and 70 countries have clear policies against it (including China, the US, and others).^24^

Nonetheless, since then, Western scientists have shown increasing openness toward human germline genome editing. A 2020 study revealed that within 5 years of Huang’s experiment, more than 10 papers have been published on the study of human germline genome editing. The ethical controversies surrounding human germline genome editing no longer exist in many countries.^25^ Similarly, a 2021 article published in the *Proceedings of the National Academy of Sciences* argued that the distinction between somatic and germline genome editing has gradually blurred since Huang’s research in 2018.^26^

Regarding human heritable genome editing, the current discussion has shifted toward exploring the priority of such research, the necessary steps before such research can occur, and how to improve the regulatory framework. The current consensus is that it is still too early to start human heritable genome editing. Even after the He Jiankui incident, some scientists, such as Shoukhrat Mitalipov and Denis Rebrikov were supporters of human heritable genome editing. Mitalipov and colleagues published a Perspective article in *Nature Medicine* in 2019, stating that heritable genome editing has great potential.^27^ Russian scientist Denis Rebrikov suggested that heritable genome editing of hearing loss genes should be used to solve congenital hearing loss, and that the decision to conduct experiments should be made by parents rather than scientists.^28^ Of course, he has not yet conducted such experiments, as he has not obtained approval from the Russian authorities. The South African Department of Health released guidelines in May 2024, with a more encouraging tone toward heritable genome editing than in other countries.^29^

After the He Jiankui incident in 2018, the NASEM of the US and the Royal Society of the UK convened members from the US, the UK, China, and seven other countries to establish the International Commission on the Clinical Use of Human Germline Genome Editing. The committee released a comprehensive report titled *Heritable Human Genome Editing* in 2020. This report highlights the importance of human heritable genome editing in avoiding genetic diseases, recommends further development of human germline genome editing, and summarizes 11 recommendations for the supervision and management of clinical translation.^30^

Even professional research societies such as the International Society for Stem Cell Research (ISSCR) revised their guidelines, and some argued that the revisions paved the way for heritable human genome editing research.^31^ For example, in 2016, ISSCR Guidelines for Stem Cell Research and Clinical Translation explicitly stated the 14-day limit for embryo manipulation, which allowed for research and some regulatory procedures on embryos without transfer to the uterus before the emergence of the primitive streak or before 14 days. In May 2021, the ISSCR revised the guidelines, recommending that national academies of science, academic societies, funders, and regulators facilitate public discussions on the possibility of extending embryo culture beyond 14 days. If broad support emerges and regulations permit within a jurisdiction, a specialized scientific and ethical oversight process could evaluate whether such research is justified. Additionally, the guidelines reclassified heritable human genome editing research from the ‘prohibited’ category to the ‘currently not permitted’ category.^32^

Many international organizations have established advisory committees to provide advisory reports to governments on national and cross-border governance. For example, the World Health Organization (WHO) established a multidisciplinary advisory committee of 18 members in 2018, which released a report on global governance standards and frameworks for human genome editing in 2021.^33^ This report elucidates the scientific value of human genome editing and proposes universal ethical principles and suggestions for global governance mechanisms to ensure that human genome editing is used responsibly and equitably.

## III. THE REGULATORY AGENCIES OF CHINA’S HUMAN GERMLINE AND HERITABLE GENOME EDITING

### III.A. The Current Status of Regulatory Agencies

**Figure 2:**
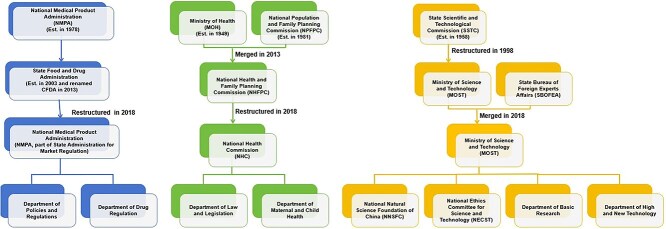
Regulatory agencies for human genome editing in China.

Figure 2 illustrates the numerous regulatory agencies involved in human genome editing, including the China National Medical Products Administration (NMPA), the National Health Commission (NHC), and the Ministry of Science and Technology (MoST).

The NHC was established during the State Council’s reform in 2018 through the restructuring of the former National Health and Family Planning Commission (NHFPC) and the former Ministry of Health (MoH). The NHC not only participates in setting guidelines and regulations for medical and healthcare practices but also governs human embryo research. The NHC has a policy department dedicated to researching laws and regulations and a women’s and children’s department that currently regulates assisted reproduction.

The NMPA was established in 1978, and in 2003, during an institutional reform, the State Food and Drug Administration (SFDA) was created, drawing on the supervisory experience of the FDA in the US. In March 2008, the direct supervisory authority of the SFDA was changed from the State Council to the MoH. In March 2013, SFDA was renamed the China Food and Drug Administration (CFDA), and, once again, became a direct subordinate agency of the State Council, no longer being managed by the MoH. In 2018, the CFDA was integrated into the State Administration for Market Regulation (SAMR), and the NMPA was re-established. The NMPA is responsible for the management of drugs, medical devices, and cosmetics in China, including the supervision of all human clinical trials involving drugs, including gene therapy products.

The MoST is responsible for the development and implementation of science and technology policies in China, the allocation of research funds, and the evaluation of scientific and technological research. It oversees research and development activities in various fields, providing guidance and regulations related to genetic research. It has a policy department, a basic research department and a high and new technology department that can regulate human genome editing. As the management body of scientific research funds in China, the National Natural Science Foundation of China (NNSFC) under the MoST is in charge of the management of national scientific research funds, and formulates national basic research guidelines, policies, and development plans. Currently, it prohibits the provision of public funds for human heritable genome editing and conducts compliance reviews for projects involving basic and preclinical research on human germline research.

Ethics committees play a crucial role in ensuring compliance with policies. In China, the former MoH established the ‘Medical Ethics Expert Committee’ (MEEC) in 2000, which was mainly responsible for formulating ethical review policies, conducting research on medical ethics, and organizing ethical training. The actual review tasks were carried out by institutional ethics committees. However, during the He Jiankui incident, the institutional ethics committee did not comply with the regulations. Professor Chen Yongchuan from the Army Medical University noted that the MEEC and regional ethics committees, which have been established for more than a decade, serve only as policy advisors. Members of institutional ethics review committees often hold multiple positions in the institution, and they temporarily serve on the committee without relevant training. There is a lack of clear administrative supervision for ethical review procedures.^34^

After the He Jiankui case, China passed the ‘Plan for the Establishment of the National Ethics Committee for Science and Technology’ in July 2019, and the MoST established the National Ethics Committee for Science and Technology (NECST) on October 22, 2021, which is focused on three areas: artificial intelligence, life sciences, and medicine.^35^ In March 2022, the General Office of the Communist Party of China Central Committee and the General Office of the State Council issued the ‘Guiding Opinions on Strengthening the Governance of Ethics of Science and Technology’.^36^ Opinions emphasize the principles of enhancing human well-being, respecting the right to life, adhering to fairness and justice, reasonably controlling risks, and maintaining openness and transparency. This means that China has recognized the importance of ethics and is using it to prevent ethical misconduct in science and health care. On July 8, 2024, the Medical Ethics Subcommittee of NECST formulated the ‘Ethical Guidelines for Human Genome Editing Research’. The Guidelines explicitly state that:

Currently, any clinical research on heritable genome editing is irresponsible and not allowed. Only when the benefits and risks, as well as other available options, are fully understood and balanced, safety and effectiveness issues are resolved, and there is a broad social consensus, can clinical research be considered after strict and prudent evaluation and under strict supervision.^37^

The new guidelines demonstrate a shift in the Chinese government’s attitude. Unlike previous policies that were more restrictive, the tone of the new guidelines is more supportive of scientific innovation. They emphasize the need to balance proactive and precautionary principles.^38^

### III.B. Comparison with Regulatory Agencies in the UK and the US

Compared with the regulatory framework led by the Human Fertilization and Embryology Authority (HFEA) in the UK and incorporating human heritable and germline genome editing into the framework of assisted reproduction, China’s regulatory framework is led by three major agencies, and the relevant policies are also mainly formulated by these three major agencies, which is closer to the joint supervision model of the National Institutes of Health (NIH) and FDA in the US.

Several European and Commonwealth countries have included human germline genome editing within the purview of assisted reproduction regulations. In the UK, the HFEA is entrusted with issuing experimental permits. The HFEA has published a comprehensive ‘HFEA Code of Practice’, which was recently updated to Version 9.3 on October 26, 2023.^39^ This code provides detailed guidelines on various techniques, such as preimplantation genetic testing, *in vitro* fertilization (IVF), fetal sex selection, gamete donation, surrogacy, mitochondrial donation, embryo storage, and transportation. For basic research on human germline genome editing, the UK and many European countries permit experiments on embryos for up to 14 days. However, the HFEA has not approved any such clinical studies yet.

The regulatory framework for human germline and heritable genome editing in the US differs from that in the UK. The US regards the application of genome editing as a therapy. Two agencies under the Department of Health and Human Services (HHS) are in charge of regulating human genome editing: the FDA and the NIH. The FDA has issued detailed institutional guidelines, providing a basis for the supervision of clinical trials involving human genome editing. These guidelines clearly explain the specific requirements and applicable standards of relevant legislation, and institutional guidelines usually solicit opinions about revisions and update regularly or irregularly. According to the ‘Human Gene Therapy Products Involving Human Genome Editing: Industry Guidelines’ released by the FDA’s Center for Biologics Evaluation and Research in January 2024, human gene therapy products should still focus on human somatic gene editing.^40^ As a result, the FDA has granted numerous investigational new drug (IND) licenses for clinical trials involving human somatic cell genome editing, but no clinical trials involving human heritable genome editing have been approved yet.

Human germline genome editing is currently conducted in laboratories, and NIH guidelines specify the biosafety practices and containment principles for these laboratories. In August 2018, NIH published a call for comments on the revision of the Recombinant DNA Guidelines in the Federal Register. In 2019, the guidelines were revised and renamed as ‘Guidelines for Research Involving Recombinant or Synthetic Nucleic Acid Molecules’.^41^ NIH requires institutions conducting any recombinant DNA research to establish an Institutional Biosafety Committee (IBC), which oversees the safe conduct of such research. The NIH Office of Science Policy (OSP) reviews and approves the membership of these committees to ensure that they have the appropriate expertise to oversee the research activities. The IBC established within the organizations is responsible for the daily supervision of biosafety and compliance with the NIH guidelines.

### III.C. Issues with Regulatory Agencies

The regulatory landscape in China regarding human genome editing is characterized by multiple agencies with overlapping responsibilities, partly due to the cutting-edge nature and complexity of the technology. Yaojin Peng and his colleagues from the CAS have pointed out that while these agencies appear to have a clear division of labor, the involvement of multiple agencies in the supervision of human genome editing has led to the diffusion of responsibilities.^42^ The overlapping responsibilities among regulatory agencies and the absence of a leading agency have resulted in regulatory lag and potential conflicts. Many have suggested that in the future, it is important to establish a regulatory agency with a clear division of responsibilities and specific functions. Professor Shi Jiayou from the Law School of Renmin University of China also pointed out that the responsibilities of these regulatory agencies overlap, and each agency acts in accordance with existing laws, which is likely to lead to lagging and decentralized responsibilities.^43^

### III.D. Recommendations for Chinese Regulatory Agencies

The first suggestion based on the principles of meticulousness and forward-looking is to separate the management of human somatic, germline, and heritable genome editing. The NMPA currently leads the supervision of products and clinical trials of human somatic genome editing. However, forward-looking discussions can be organized among policymakers, legal and ethical experts to determine whether the NHC, the NMPA, or the MoST will take the leading role in managing human heritable genome editing in the future. This study suggests that the NHC take the lead in management. There are three reasons for this: First, the NHC is already responsible for managing numerous clinics in China that provide IVF services. Second, clinical trials or therapies involving heritable genome editing are more complex than drugs are. They require rigorous evaluation of the blastocyst before implantation and continuous monitoring during pregnancy. In addition, both children and adults require long-term follow-up after birth. Third, other assisted reproductive technologies are being developed and require long-term supervision and regulation. For example, a more advanced technology than human heritable genome editing is *in vitro* gametocytosis, which is still in the basic research stage but may have a revolutionary impact on infertility treatment and traditional egg retrieval processes. It is more advantageous to incorporate all these new technologies into the framework of assisted reproduction rather than gene therapy.

The second suggestion based on the principle of balance is that for human germline genome editing which does not involve clinical trials or new drug development, reasonable regulation should be adopted, but there should be no excessive regulation. The MoST can serve as the leading department responsible for such supervision. As the management agency of China’s largest scientific research fund, the NNSFC under the MoST should strengthen the management of research funds. At present, providing public funds for human heritable genome editing is strictly prohibited by the NNSFC, and compliance reviews should be conducted for human germline genome editing. The newly established NECST under the MoST can also play a key role in helping formulate policies, providing advisory reports, promoting public participation, and assisting in the review of experimental research.

The third suggestion, which is based on the principle of adaptability, is that regulatory agencies should have a mechanism to assist in regularly updating policies. Regulatory agencies assist legislators in issuing specialized laws or detailed guidelines to prevent the publication of scattered legal provisions and organize experts to evaluate the legal effects regularly.

## IV. POLICIES REGARDING HUMAN GERMLINE AND HERITABLE GENOME EDITING IN CHINA

### IV.A. Current Status of Policies in China

**Figure 3:**
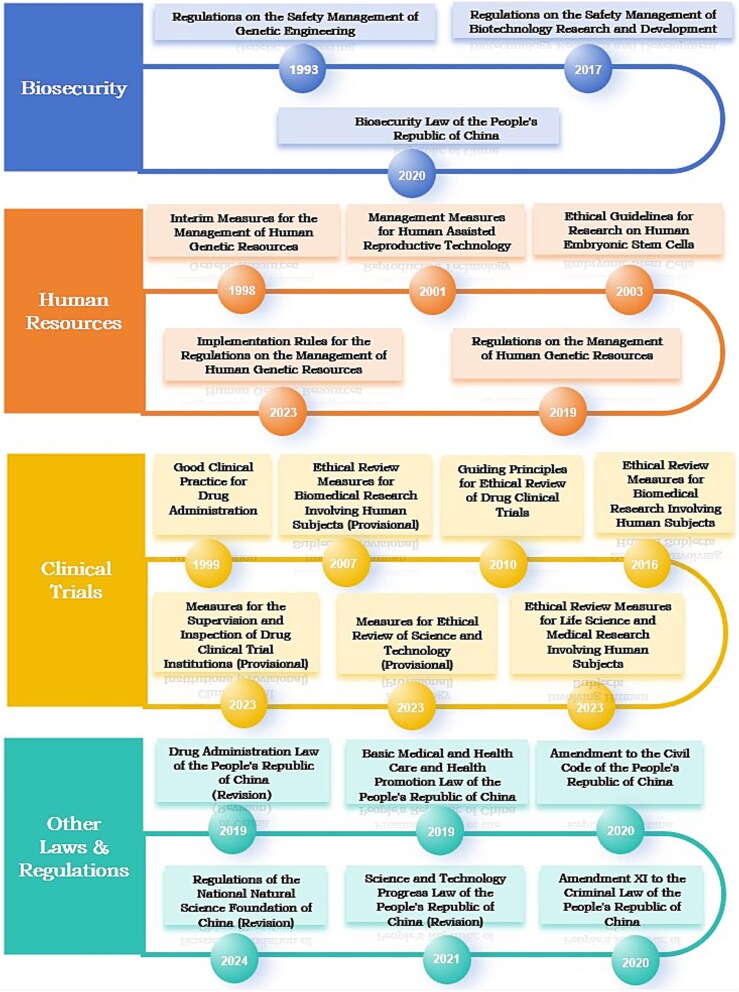
Policies related to human genome editing in China.

Over the past 20 years, China has drafted and revised several laws and regulations concerning human genome editing, as depicted in Figure 3. Overall, the Chinese legislators responded quickly to the He Jiankui incident, by issuing new laws and regulations or revising existing ones. Second, violations related to human genome editing were often included in low-level administrative regulations, and the He Jiankui incident promoted the inclusion of such violations in laws.^44^ For example, in the past, most ethical review policies for drug clinical trials in China were administrative regulations, such as the ‘Good Clinical Practice for Drug Administration’ in 1999, which were later written into the revised *Drug Administration Law* for the first time in 2019. Article 20 clearly stipulates that ‘conducting clinical trials shall comply with ethical principles, develop clinical trial plans, and obtain approval from the ethics committee’. In addition, in terms of legal research, China has taken several measures to promote the improvement of regulatory policies by funding relevant research projects and organizing seminars on legal regulation of human genome editing by the Ministry of Justice (MoJ). For example, in 2019 alone, the MoJ funded five research projects related to human genome editing policies.^45^ These projects reflect the emphasis on collaborating with academia to improve policies.

In China, human genome editing is regulated by four main types of policies. The first category is related to biosafety. As early as 1993, China issued the ‘Regulations on the Safety Management of Genetic Engineering’. These regulations cover aspects such as approval, supervision, safety assessment, and accident management of genetic engineering projects, aiming to prevent potential risks that genetic engineering may pose to the environment and human health. These regulations focus more on biosafety rather than ethical review.^46^ In 2017, the MoST issued the ‘Regulations on the Safety Management for Biotechnology Research and Development’. China passed the *Biosecurity Law* in 2020, which clarifies the legal responsibilities for violations. For example, individuals engaged in biological research prohibited by law are subject to clear penalties such as fines and revocation of professional certificates.^47^

The second category of policy is related to human genetic resources, assisted reproductive technology, and human embryonic stem cell research. As early as 1997, geneticist Tan Jiazhen wrote a letter to the central government calling for the protection of human genetic resources in China. Tan’s letter has attracted attention from the central government, and the MoST and the former MoH have jointly formulated the ‘Interim Measures for the Management of Human Genetic Resources’. According to the Measures, the MoST established the ‘Management Office of Chinese Human Genetic Resources’ in 1999, which is mainly responsible for regulating and managing the collection, trade, export, and other matters related to Chinese human genetic resources. The former MoH also promulgated and implemented the ‘Management Measures for Human Assisted Reproductive Technology’ as early as 2001, which prohibits situations involving major ethical disputes in assisted reproductive technology, including buying and selling reproductive cells, surrogacy, gender selection, etc. Medical institutions must establish medical ethics committees and provide informed consent when assisted reproductive technology is implemented. In 2003, the MoST and the former MoH jointly issued the ‘Ethical Guidelines for Research on Human Embryonic Stem Cells’, emphasizing the 14-day rule for embryonic research, explicitly prohibiting human cloning research, and stipulating that institutions involving human embryonic stem cell research should establish ethics committees. In January 2016, the MoST submitted a draft of the ‘Regulations on the Management of Human Genetic Resources’ to the former Legislative Affairs Office of the State Council. After multiple public solicitations of opinions and revisions by the MoJ and the MoST, on May 28, 2019, Premier Li Keqiang signed a State Council order and officially promulgated the ‘Regulations on the Management of Human Genetic Resources’, which regulate the use of Chinese human genetic resources by foreign and domestic institutions and individuals, to ensure that these activities do not endanger China’s public health and safety, and include administrative penalties for violations.^48^ After the promulgation of the Regulations, the MoST extensively solicited experts’ opinions, launched the formulation of relevant supporting implementation rules, and issued the detailed ‘Implementation Rules for the Regulations on Human Genetic Resources’ in May 2023.

The third category is related to the ethical review of China’s biomedical research and clinical trials, which China has vigorously promoted in the past two decades. First, as China continues to invest in the R&D of biomedical technology, establishing and improving ethical review systems has become increasingly important. Second, the development of medicine with international standards has promoted the standardization of medical ethics reviews. In 2007, the former MoH issued the ‘Ethical Review Measures for Biomedical Research Involving Human Subjects (Provisional)’, and in 2016, the former NHFPC officially issued the ‘Ethical Review Measures for Biomedical Research Involving Human Subjects’. The measures supplement previous regulations to ensure that research in sensitive areas such as human germline genome editing is not only regulated in terms of biosafety but also complies with detailed ethical standards. These measures clarify the scope of application, basic principles, and responsibilities of ethical committees. It requires medical and healthcare institutions at all levels and relevant research institutions to establish ethics committees, which review content, including informed consent, risk control, privacy protection, etc., to ensure the ethical standard of research. In addition, the measures clearly stipulate that institutions and individuals who violate the provisions of these measures and damage the personal property of others will bear civil liability or be held criminally responsible in accordance with the law. Similarly, in 2010, the former SFDA issued the ‘Guiding Principles for Ethical Review of Drug Clinical Trials’. After the He incident, in February 2023, the NHC, together with the Ministry of Education, the MoST, and the State Administration of Traditional Chinese Medicine, released the revised ‘Ethical Review Measures for Life Science and Medical Research Involving Human Subjects’. The Measures have expanded the scope of ethical review and are more targeted toward new technologies, such as gene editing, information technology, and modern reproductive technology. In September 2023, the MoST issued the ‘Measures for Ethical Review of Science and Technology (Provisional)’, and on November 3, 2023, the NMPA issued the ‘Measures for the Supervision and Inspection of Drug Clinical Trial Institutions (Provisional)’.

The fourth category includes other common laws and regulations related to health care, science and technology, as well as civil and criminal laws, such as the *Drug Administration Law*, *Science and Technology Progress Law*, *Basic Medical and Health Care and Health Promotion Law*, and the ‘Regulations of the National Natural Science Foundation of China’. Following He Jiankui’s case, in May 2020, the *Civil Code* added a new article about medical and scientific research activities related to human genomes and embryos.^49^ In December 2020, *Amendment XI of the Criminal Law of the People’s Republic of China* added the crime of ‘illegal genome editing and cloning of embryos’.^50^ Another example is the *Science and Technology Progress Law*, which was promulgated and implemented in 1993, revised for the first time in 2007, and passed its second revision in January 2022, emphasizing the establishment and improvement of scientific research integrity and ethics management systems.

### IV.B. Comparison with Policies in the UK and the US

In terms of legislation, China is closer to the US mode rather than to the UK mode. Several countries have implemented special legislation in the field of assisted reproduction. These legislations currently prohibit clinical trials involving human heritable genome editing. For example, the UK passed the *Human Fertilization and Embryology Act* in 1990, which was amended in 2008. The regulation of human genome editing in the US relies on several policies, including the *Public Health Service Act*, the *Food, Drug, and Cosmetic Act*, and those related to federal appropriations and patents.^51^ According to an appropriation bill rider known as the ‘Dickey-Wicker Amendment’, federal appropriations cannot be used to create, destroy, or discard human embryos for research purposes or knowingly place embryos in the human uterus.^52^ NIH has emphasized that NIH funding does not support human germline genome editing. However, basic experiments on human germline genome editing can be carried out in certain states, for example, using private funds. Clinical trials, on the other hand, require FDA approval and are thus subject to the *Food, Drug, and Cosmetic Act* and FDA guidelines on gene therapy. This legislative approach, which is based on project funding, biological products, and patents, helps determine the direction of research and ensures proper guidance.

### IV.C. Issues with Policies in China

However, several issues with China’s current laws and regulations remain. First, many laws and regulations in this domain have not been updated promptly. For example, the ‘Management Measures for Human Assisted Reproductive Technology’ was issued by the MoH in 2001 and has not been revised for more than 20 years. Similarly, the ‘Ethical Guidelines for Research on Human Embryonic Stem Cells’ was issued jointly by the MoST and the MoH in 2003 and has not been updated for nearly 20 years. Second, many of these policies are administrative regulations rather than laws, and punishments for violations are often limited to administrative notices of criticism or warnings, without clear definitions of charges and corresponding legal consequences. For example, although He Jiankui blatantly violated the provision in the 2003 ‘Ethical Guidelines for Research on Human Embryonic Stem Cells’ that prohibits clinical trials involving human heritable genome editing, the violation did not entail specific charges or sentences. Third: although many relevant laws and regulations have undergone some revisions, the current laws often lack specificity, particularly in categorizing human genome editing. It is crucial to treat basic and preclinical experiments and clinical trials differently, as well as distinguish between somatic, germline, and heritable genome editing.

### IV.D. Recommendations for Chinese Legislation

At present, a key question in China’s legislation regarding human heritable and germline genome editing is whether to legislate specifically or to de-specialize it. Should China enact a specialized law, or integrate it into existing policies and provide more detailed revisions? Professor Shi Jiayou noted that the existing legal system is very complex and suggested issuing a specialized law such as the *Human Fertilization and Embryology Act*.^53^ This legislative approach is closer to the UK model.

However, this approach has several limitations. First, China’s regulatory model is closer to that of the US, with multiple agencies overseeing rather than a single agency being responsible, and several laws and regulations governing together. Second, how can the degree of specialization be determined? Should the specialized law focus on human heritable and germline genome editing, or human genome editing, including somatic cell editing, or even genome editing technology in all organisms, including animals and plants?

Based on the principle of forward-looking, considering the possibility of multiple new reproductive technologies emerging in the future, we propose that human genome editing should be regulated in the context of other emerging technologies in the life sciences, such as synthetic biology and nanotechnology. This article calls for the de-specialization of human germline and heritable gene editing and its integration into existing regulatory systems. For example, in the US, biotechnology regulation is guided by the Coordinated Framework for Regulation of Biotechnology, first issued in 1986.^54^ This framework emphasizes the use of existing regulatory agencies—such as the FDA, the Environmental Protection Agency (EPA), and the US Department of Agriculture (USDA)—to oversee biotechnology products, rather than establishing a new regulatory agency specifically for biotechnology. According to *Heritable Human Genome Editing* edited by the National Academy of Medicine, the National Academy of Sciences, and the Royal Society, human heritable genome editing can be integrated into two frameworks. One is to incorporate it into the regulatory framework of gene therapy, which also regulates the clinical trials of human somatic gene editing. Currently, such trials are more common in China. For example, China approved the world’s first gene therapy product, *Gendicine*, as early as 2003. Another is the regulatory framework for assisted reproductive technology. China has relevant policies in both fields.

Based on the principle of meticulousness, a classified management approach should be adopted for human genome editing. When formulating policies, China needs to establish clear standards for different types of basic research, preclinical research, and clinical trials related to human gene editing. Different standards should also be applied for gene enhancement and gene therapy.

Based on the principle of adaptability, legislators and policy makers should regularly revise laws and regulations, in line with advancements in high-tech development and the current practical needs of the country. When formulating new laws and regulations, it is necessary to consult extensively with experts in the field to ensure that the legislation is carefully considered. The WHO recommends holding regular meetings at least every 3 years to review and update the governance framework for human genome editing.

Based on the principle of balance, excessive regulation should be avoided and a balance between safety and innovation should be sought. For example, in assisted reproduction, surrogacy is currently prohibited in China. However, as Professor Zhu Zhen from Jilin University Law School noted, overly evasive and conservative policies can lead to the emergence of an enormous underground market.^55^ Currently, some scholars have suggested allowing altruistic surrogacy and commercial surrogacy due to the widespread demand in China and the pressure of an aging population and low birth rates.^56^ The authors are not calling for the relaxation of clinical research on human heritable gene editing, but rather pointing out that the law is not static and needs to keep up with the development of the times.

## V. THE INVOLVEMENT OF MULTIPLE SOCIAL GROUPS RELATED TO THE HUMAN GERMLINE AND HERITABLE GENOME EDITING IN CHINA

### V.A. Current Status and Issues of the Involvement of Multiple Social Groups in China

Human germline and heritable genome editing is an emerging technology that requires the involvement of various social groups in addition to the government authorities. It is crucial to include the opinions of intergovernmental organizations (such as the WHO), scientific organizations (such as the CAS), ethical organizations, think tank groups, the media, scientists and medical practitioners at stake, patient advocacy groups, and the public in the decision-making process.

The literature review of this article discussed many academic groups related to the He Jiankui incident, including the WHO, the NASEM, the CAS, Chinese ethics scholars, etc. The following section emphasizes the perspective of public and stakeholder engagement (PSE), ie, the scientists and medical professionals engaged in human genome editing, as well as patient groups and the public.

Before the He Jiankui incident, the Chinese public was generally in favor of human genome editing. Research shows that the Chinese public usually holds a liberal attitude toward human heritable genome editing if it helps protect infants from genetic diseases.^57^ With respect to genetic enhancement, Wang et al. (2017) conducted an online public survey about the attitudes of Chinese people and found that survey participants were more willing to conduct genetic enhancement for nonmedical reasons than clinicians were.^58^

After the He Jiankui incident, according to a research conducted by Zhang Juanjuan and her colleagues in 2019, 47.7% of the Chinese public aged 16 years and above still supported the application of gene therapy and genetic enhancement, 27.7% supported only gene therapy, 1.9% supported only genetic enhancement, and 22.7% opposed both genetic therapy and gene enhancement. They noted that less than 20% of the participating public had a detailed understanding of the He Jiankui incident.^59^ The other reasons why the Chinese public generally supports genetic technology may be that they are not influenced by religion, and unlike eugenics, which has a negative connotation in the Western context, the Chinese public generally accepts the concept of eugenics. The Chinese public often turns to social media to seek information and share opinions on public issues they are concerned about. Zhang Xing and colleagues from the National University of Singapore investigated public discussions on this matter on the social platform Sina Weibo. Their research revealed that a significant number of Weibo users criticized the inadequate regulation of genetic research and called for immediate legislation in China.^60^

However, Lei Yawen from the Department of Sociology at Harvard University noted that in China, owing to the imbalance of knowledge between the public and experts, the public is often excluded from policy decisions.^61^ Some scientists oppose increasing public discussion on human genome editing issues in China, because they believe that the public is ignorant and that the Chinese public’s ability to influence policies, legislation, regulation, and research funding is limited.

There are also areas for improvement in the participation of professional groups. First, there is a lack of understanding among Chinese scientists regarding the popularization of science. Owing to being busy or for other reasons, frontline scientists and related professionals are less involved in such activities. He Jiankui is a counterexample. He was interviewed at the Second International Summit on Human Gene Editing, but it was believed to be for commercial purposes. Scientists in China are considered to be more low-key and focus on conducting research. Wang Dapeng and his colleagues from the China Institute of Science Popularization believe that Chinese scientists have four types of unwillingness toward science popularization, namely unwillingness, disdain, fear, and lack of expertise.^62^

The He Jiankui incident revealed a lack of ethical education among medical practitioners, ethical review committees, and regulatory authorities in China. Professors Xu Tianxu and Shi Yumin from the University of Science and Technology of China noted that there is a significant gap in terms of teaching staff and educational resources for ethics education at Chinese universities. Many universities lack a team of ethics teachers, making offering ethics courses difficult.^63^ In contrast, some foreign universities place great importance on ethics courses. In the spring of 2019 alone, Harvard University School of Medicine offered 16 courses on the ethics of technology. Chinese universities should provide more training on such courses for medical students, reproductive clinic practitioners, and scientists. Professor Zhang Di from the Chinese Academy of Medical Sciences and his colleague believe that there are few opportunities for interaction between Western and Chinese researchers and bioethicists. Language barriers are an obvious reason, but the lack of a sustained institutional framework for interaction between Chinese bioethicists and their Western counterparts is also an important reason.^64^

### V.B. Experience from the Abroad

Think tanks play a crucial role in shaping the regulatory framework for genome editing, particularly in representing the voices of the public and patient groups. In the UK, the Nuffield Council on Bioethics, with over 25 years of experience, provides valuable insights for enhancing think tanks’ participation in developing regulations for human genome editing. The Council organizes expert working groups and conducts multiple investigations, leading to the publication of significant reports on genome editing ethics, including *Genome Editing: An Ethical Review* published in 2016,^65^ and *Genome Editing and Human Reproduction: Social and Ethical Issues* published in 2018.^66^ The Council emphasizes public participation and advocates for the protection of vulnerable groups with disabilities. It recognizes that the value of these reports lies in fostering further dialogue and exploration. Striking a balance between professionalism and public opinion is essential, ensuring that the legislative process considers the reasonable demands of the general public while also incorporating expert insights. In 2018, the committee conducted a public investigation, held meetings with experts in relevant fields including developmental biology, law, and bioethics, and interviewed patient advocacy groups. The Council emphasized the concept of PSE, actively involving those who may be affected (such as those with genetic diseases, their parents and family members, couples who may have children with severe genetic diseases) and those affected by inequality.^67^ Similarly, the NASEM and the Royal Society widely adopted public suggestions when leading the preparation of their 2020 report, holding two public meetings (in Washington and London), four public webinars and lectures, and opening up public comment sections and live video exchanges.^68^

In the US, national-level bioethics commissions have played significant roles in advising administrations on ethical issues related to biomedical science and technology from the late 1970s through the end of the Obama administration. However, no similar commission has been established under either the Trump or Biden administrations.^69^ The German Ethics Committee, the German Academy of Sciences, the Indian Medical Research Council, the Dutch Genetic Modification Commission, the Japanese Scientific Committee, and the Royal Society of New Zealand have issued important reports on human genome editing in recent years.^70^ China can fully draw on the experience of such committees and entrust institutions such as the newly founded NECST to form committees to issue similar reports, which will become an important reference for the government’s regulatory agencies. Although the NECST was established under the leadership of the MoST, the committee is led mainly by academics, so it can also undertake responsibilities similar to those of the Nuffield Bioethics Committee. For example, in its latest guidelines, the NECST emphasizes both the principle of caution and the principle of proactivity in a balanced manner, which is more supportive of research and innovation than some laws and regulations in previous years.^71^

In the UK, the patient population has played a crucial role in advocating for the approval of mitochondrial replacement technology during its translation process.^72^ Professionals frequently spoke on behalf of patient groups in public, but later, more and more patients actively began to speak out, sharing their own experiences and those of their families. After extensive scrutiny, including scientific reviews, public consultations, listening to patients’ voices, and debates in Parliament, the UK legislated to approve this technology.

### V.C. Recommendations for the Involvement of Various Social Groups in China

**Figure 4:**
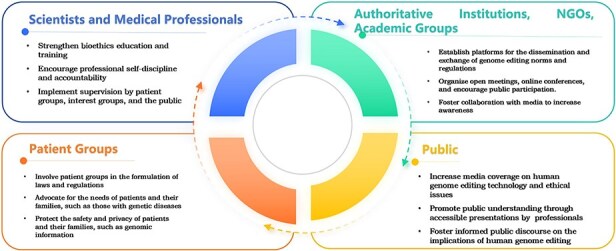
Recommendations for the participation of multiple social groups related to human genome editing.

Figure 4 illustrates the recommendations for the participation of Chinese social groups in human heritable and germline genome editing. The following specific measures can help improve the participation of these groups.

First, strengthening bioethics education and training for scientists and medical professionals with vested interests is fundamental for alleviating regulatory pressure, providing practitioners with the correct values, and enhancing their sense of social responsibility. Professional self-discipline can be an effective way to hold scientists accountable. The process of self-discipline may also include supervision by representatives of patient groups, interest groups, and the public. For example, the guidelines developed by the ISSCR have had a strong impact on national and international policies and regulations.^73^

Second, authoritative institutions, non-governmental organizations, academic groups including scientific, ethical, and legal professionals, and media should establish platforms for the exchange and dissemination of genome editing norms and regulations, allowing individuals from all backgrounds to contribute to the regulatory framework. For example, policy-making agencies of various government departments can increase communication with the aforementioned organizations, hold open meetings or online conferences, and encourage public participation.

Third, media coverage of genome editing technology and its ethical issues can enhance public awareness and understanding. On various platforms, the public can express their opinions and deepen their understanding and knowledge of genome editing technology. Scientists also have a responsibility to give speeches to the public to promote their understanding.

Finally, engaging in dialogue with patient groups. Whether it is intergovernmental organizations, scientific organizations, media, or practitioners, they should focus on patient groups and consider their risks and benefits, not only to meet their various needs, but also to think about regulatory frameworks that can protect their safety and avoid a repeat of the He Jiankui incident or the tragedy of Jess Gelsinger’s death due to gene therapy in the US in 1990. Patient advocacy groups’ articles can be published on news, social media, and other channels, and patient groups should be involved in the formulation of laws and regulations to ensure openness and transparency, as suggested by the WHO^74^. In addition, it is necessary to protect the privacy rights of patients and their family members and strengthen the protection of their genomic information.

## VI. CONCLUSIONS

The biotechnology industry is a rapidly growing and globalized sector, often referred to as the ‘Sunrise Industry’ of the 21st century in China. Due to its high technology, high investment, high risk, and high efficiency, countries worldwide consider the biotechnology industry to be a key sector for economic development. Although China’s biotechnology industry started relatively late, it has demonstrated strong momentum. The inclusion of genetic engineering technology in China’s medium to long-term scientific and technological development plans reflects the importance given by relevant national bureaus to genetic engineering. Human genome editing technology is one of the most important and disruptive technologies and has significant economic and social value.

On the other hand, science is not value-free, and science and technology cannot be separated from the cultural contexts of different countries. The role of policy guidance is crucial for the rapid advancement of China’s biotechnology industry on the global stage. Given its implications for national health and significant societal benefits, China should develop a forward-looking, balanced, meticulous, and adaptable regulatory approach. This article suggests that national regulatory agencies should clarify their responsibilities, establish policies that classify human genome editing, and promote dialogue among ethicists, scientists, legislators, patient groups, and the public.

## Footnotes

1 Organising Committee of the Third International Summit on Human Genome Editing, *Statement from the Organising Committee of the Third International Summit on Human Genome Editing*, https://royalsociety.org/news/2023/03/statement-third-international-summit-human-genome-editing/ (accessed Dec. 1, 2024).

2 Alex Philippidis, *CASGEVY Makes History as FDA Approves First CRISPR/Cas9 Genome Edited Therapy*, 35 Hum. Gen. The. 1 (2024).

3 Françoise Baylis et al., *Human Germline and Heritable Genome Editing: The Global Policy Landscape*, 3 Crispr J. 365 (2020).

4 Matthew P. Hirakawa et al., *Gene Editing and CRISPR in the Clinic: Current and Future Perspectives*, 40 Biosci. Rep. Bsr 20200127 (2020).

5 Michael Morrison & Stevienna de Saille, *CRISPR in Context: Towards a Socially Responsible Debate on Embryo Editing*, 5 Palgrave  Commun. 110 (2019).

6 Dennis Normile, *China Sprints Ahead in CRISPR Therapy Race: Human Trials are Using the Genome-Editing Technique to Treat Cancers and Other Conditions*, 358 Science 20 (2017).

7 Yuyu Niu et al., *Generation of Gene-Modified Cynomolgus Monkey via Cas9/RNA-Mediated Gene Targeting in One-Cell Embryos*, 156 Cell 836 (2014).

8 David Cyranoski, *CRISPR Gene-Editing Tested in a Person for the First Time*, 539 Nature 479 (2016).

9
ClinicalTrials.gov. *Clinical Trials Employing Human Somatic Cell Genome Editing*, https://clinicaltrials.gov/ct2/results?term=crispr&map_cntry=CN (accessed July 17, 2023).

10 Lei Xu et al., *CRISPR-Edited Stem Cells in a Patient with HIV and Acute Lymphocytic Leukemia*, 381 N. Engl. J. Med. 1240 (2019).

11 Songyang Zhang et al., *Current Trends of Clinical Trials Involving CRISPR/Cas Systems*, 10 Front. Med.1292452 (2023).

12 Puping Liang et al., *CRISPR/Cas9-Mediated Gene Editing in Human Tripronuclear Zygotes*, 6 Protein  Cell 363 (2015).

13 Renzhong Qiu, *Research and Application of Genome Editing Technology: An Ethical Perspective*, 37 Med. Philos. [Yixue  Yu  Zhexue] 1 (2016).

14 Didi Kirsten Tatlow, *Scientific Ethical Divide Between China and West*, The  New  York  Times (Health & Medicine), June 29, 2015.

15 Edward Lanphier et al., *Don’t Edit the Human Germ Line*, 519 Nature 410 (2015).

16 Jocelyn Kaiser & Dennis Normile, *Embryo Engineering Study Splits Scientific Community*, 348 Science 486 (2015).

17 ANON, *365 Days: Nature’s 10: Ten People Who Mattered This Year*, Nature, Dec. 15, 2015, http://www.nature.com/news/365-days-nature-s-10-1.19018 (accessed July 17, 2023).

18 National Academy of Sciences, Engineering and Medicine, *International Summit on Human Gene Editing: A Global Discussion*, https://nap.nationalacademies.org/download/21913 (accessed July 17, 2023).

19 Committee on Human Gene Editing: Scientific, Medical, and Ethical Considerations, *Human Genome Editing: Science, Ethics, and Governance* 6 (2017).

20 Organizing Committee of the Second International Summit on Human Genome Editing, *Statement by the Organizing Committee of the Second International Summit on Human Genome Editing*, http://www.nationalacademies.org/news/2018/11/statement-by-the-organizing-committee-of-the-second-international-summit-on-human-genome-editing (accessed July 17, 2023).

21 Eric S. Lander et al., *Adopt a Moratorium on Heritable Genome Editing*, 567 Nature 165 (2019).

22 Ruipeng Lei et al., *Reboot Ethics Governance in China*, 569 Nature 184 (2019).

23 Renzong Qiu et al., *Ethical and Governance Challenges Arising from the Editing of Heritable Genomes*, 4 Med. Philos. [Yixue  Yu  Zhexue] 1 (2019).

24 Baylis, *supra* note 3, at 367.

25 Emilia Niemiec & Heidi Carmen Howard, *Ethical Issues Related to Research on Genome Editing in Human Embryos*, 18 Comput. Struct. Biotechnol. J. 887 (2020).

26 John H. Evans, *Setting Ethical Limits on Human Gene Editing After the Fall of the Somatic/Germline Barrier*, 118 Proc. Natl. Acad. Sci. U. S. A. e2004837117 (2021).

27 Don P. Wolf et al., *Principles of and Strategies for Germline Gene Therapy*, 25 Nat. Med. 890 (2019).

28 David Cyranoski, *Russian ‘CRISPR-Baby’ Scientist Has Started Editing Genes in Human Eggs with Goal of Altering Deaf Gene*, 574 Nature 465 (2019).

29 Donrich Thaldar et al., *Human Germline Editing: Legal-ethical Guidelines for South Africa*. 116 S. Afr. J. Sci. 1 (2020).

30 International Commission on the Clinical Use of Human Germline Genome Editing, National Academy of Medicine, National Academy of Sciences, The Royal Society, *Heritable Human Genome Editing* 6 (2020).

31 Françoise Baylis, *Heritable Human Genome Editing is ‘Currently not Permitted’, But It Is No Longer ‘Prohibited’: So Says the ISSCR*, 49 J. Med. Ethics 319 (2023).

32 ISSCR, *Guidelines for Stem Cell Research and Clinical Translation*, http://www.isscr.org/guidelines (accessed Oct. 10, 2022).

33 WHO Expert Advisory Committee on Developing Global Standards for Governance and Oversight of Human Genome Editing, *Human Genome Editing: A Framework for Governance* 1 (2021).

34 Yongchuan Chen, *Review and Outlook: The Development Trend of Ethical Review in Biomedical Research in China*, 8 Med. Philos. [Yixue  Yu  Zhexue] 1 (2020).

35 Jia Ping, *From the Perspective of the Operational Mechanisms of Major National Bioethics Committees around the World, the Governance of Technological Ethics.* 1 China  Reform [Zhongguo  Gaige] 79 (2022).

36 Ge Haitao, *The Process and Prospect of Ethical Governance in Science and Technology in China.* 4 Today’s  Sci. Tech. Gard. [Jinri  Keyuan] 32 (2023).

37 Ministry of Science and Technology of China, *Ethical Guidelines for Human Genome Editing Research*, https://www.most.gov.cn/kjbgz/202407/t20240708_191311.html (accessed July 23, 2024).

38 Joy Zhang, *China’s Ethics Guidelines – A New Era for Human Genome Editing?,*  BioNews, Aug. 12, 2024, at 1.

39 HFEA, *The Code of Practice version 9.4*, https://www.hfea.gov.uk/about-us/news-and-press-releases/2023/the-code-of-practice-version-94-is-now-available/ (accessed May 2, 2024).

40 Center for Biologics Evaluation and Research, *Human Gene Therapy Products Incorporating Human Genome Editing: Industry Guidelines*, https://www.fda.gov/regulatory-information/search-fda-guidance-documents/human-gene-therapy-products-incorporating-human-genome-editing (accessed Feb. 13, 2024).

41 National Institutes of Health, *Final Action under the NIH Guidelines for Research Involving Recombinant or Synthetic Nucleic Acid Molecules (NIH Guidelines)*, https://www.federalregister.gov/documents/2019/04/26/2019-08462/final-action-under-the-nih-guidelines-for-research-involving-recombinant-or-synthetic-nucleic-acid (accessed Jan. 15, 2024).

42 Yaojin Peng et al., *Responsible Governance of Human Germline Genome Editing in China*, 107 Biol. Reprod. 261 (2022).

43 Shi Jiayou & Liu Zhongxuan, *Risk Response of Gene Editing Technology: Ethical Governance and Legal Regulation*, 1 Rule  Law  Res. [Fazhi  Yanjiu] 86 (2023).

44 Zhang Xiaoxiao, *Research on the Ethical and Legal Coordination Mechanism of Offspring from the Perspective of Reproductive Genome Editing*, 45 Dial  Nat  News [Ziran  Bianzhengfa  Tongxun] 88 (2023).

45 Ministry of Justice, *Announcement on the Approval of the 2019 National Rule of Law and Legal Theory Research Project*, http://www.moj.gov.cn/pub/sfbgw/zwxxgk/fdzdgknr/fdzdgknrtzwj/201911/t20191128_373128.html (accessed Dec. 28, 2019).

46 Shi Jiayou & Zeng Jia, *International Experience and Chinese Model of Biosafety Legal Governance*, 3 Academic [Xueshujie] 53 (2024).

47 Leifan Wang et al., *Human Genome Editing after the “CRISPR Babies”: The Double-Pacing Problem and Collaborative Governance*, 5 J. Biosaf. & Biosecurity 8 (2023).

48 Shi & Liu, *supra* note 43, at 88.

49 The 13th National People’s Congress of the People’s Republic of China, *Civil Code of the People’s Republic of China, Article 1009*, http://www.npc.gov.cn/npc/c30834/202006/75ba6483b8344591abd07917e1d25cc8.shtml (accessed July 17, 2023).

50 The 13th National People’s Congress of the People’s Republic of China, *Amendment (XI) to the Criminal Law of the People’s Republic of China, Article 336*, https://flk.npc.gov.cn/detail2.html?ZmY4MDgxODE3OTZhNjM2YTAxNzk4MjJhMTk2NDBjOTI (accessed July 17, 2023).

51 Morgan Mendicino, *Genetically Customized Generations—A Need for Increased Regulatory Control Over Gene Editing Technology in the United States*, 73 SMU Law  Rev. 585 (2020).

52 House of Representatives, *H.R. 2880*, http://thomas.loc.gov/cgi-bin/bdquery/z?d104:H.R.2880 (accessed July 17, 2023).

53 Hu Xinping & Shi Jiayou, *The Problems and Improvement of the Governance Framework of Human Germline Genome Editing in China*, 1 Econ. Trade L. Rev. [Jingmao  Falv  Pinglun] 30 (2022).

54 Executive Office of the President, Office of Science and Technology Policy, *Coordinated Framework for Regulation of Biotechnology*, https://www.aphis.usda.gov/sites/default/files/coordinated_framework.pdf (accessed Mar. 25, 2025).

55 Zhu Zhen, *Against Perfection: Moral and Legal Philosophical Reflections on Human Genome Editing*, 21 J. Ecupl [Huadong  Zhengfa  Daxue  Xuebao] 72 (2018).

56 Zhang Lijun, *A Brief Analysis of the System Design of Surrogacy in China from “Blocking” to “Relaxing,”* 31 China  Health L. [Zhongguo  Weisheng  Fazhi] 70 (2023).

57 Di Zhang & Reidar K. Lie, *Ethical Issues in Human Germline Gene Editing: A Perspective from China*, 36 Monash  Bioeth. Rev. 23 (2018).

58 Jianghui Wang et al., *Public Attitudes Toward Gene Therapy in China*, 6 Mol. Ther., Methods  Clin. Dev. 40 (2017).

59 Juanjuan Zhang et al., “*Gene Therapy” or “Gene Enhancement”: The Public’s Acceptance of Gene Editing Technology and Its Influencing Factors*, 13 Sci. & So. [Kexue & Shehui] 107 (2023).

60 Xing Zhang et al., *Before and After the Chinese Gene-Edited Human Babies: Multiple Discourses of Gene Editing on Social Media*, 30 Public  Underst. Sci. 570 (2021).

61 Yawen Lei, *Publics, Scientists, and the State: Mapping the Global Human Genome Editing Controversy*, 246 China Q. 400 (2021).

62 Wang Dapeng et al., *Thinking about the Current Situation and Path of Scientists’ Participation in Science Popularization in the New Era*, 11 J. Cas. [Zhongguo  Kexueyuan  Yuankan] 1994 (2024).

63 Tianxu Xu & Yumin Shi, *Foreign Reference and Enlightenment on Science and Technology Ethics Education in Universities*, 31 J. Chang. Univ.[Changchun  Daxue  Xuebao] 51 (2021).

64 Zhang & Lie, *supra* note 57, 24.

65 Nuffield Council on Bioethics, *Genome Editing: An Ethical Review*, https://www.nuffieldbioethics.org/publications/genome-editing-an-ethical-review (accessed July 17, 2023).

66 Nuffield Council on Bioethics, *Genome Editing and Human Reproduction: Social and Ethical Issues*, https://www.nuffieldbioethics.org/publications/genome-editing-and-human-reproduction (accessed July 17, 2023).

67 Wendy P. Geuverink et al., *The Need to Set Explicit Goals for Human Germline Gene Editing Public Dialogues*, 1 J. Community  Genet. 7 (2024).

68 Ana S. Iltis et al., *Public and Stakeholder Engagement in Developing Human Heritable Genome Editing Policies: What Does It Mean and What Should It Mean?*, 3 Frontiers  Pol. Sci. 730869 (2021).

69 Christine Grady, *Making the Choices Necessary to Make a Difference: The Responsibility of National Bioethics Commissions*, 47 Hastings  Cent. Rep. S42 (2017).

70 Xu & Shi, *supra* note 63, at 52.

71 Zhang, *supra* note 38, at 1.

72 Rebecca Dimond & Neil Stephens, *Three Persons, Three Genetic Contributors, Three Parents: Mitochondrial Donation, Genetic Parenting and the Immutable Grammar of the ‘Three X X’*, 22 Health 240 (2018).

73 WHO, *supra* note 33, at 41.

74 WHO, *supra* note 33, at x.

